# Pharmacogenetics in the Treatment of Cardiovascular Diseases and Its Current Progress Regarding Implementation in the Clinical Routine

**DOI:** 10.3390/genes10040261

**Published:** 2019-04-01

**Authors:** Cristina Lucía Dávila-Fajardo, Xando Díaz-Villamarín, Alba Antúnez-Rodríguez, Ana Estefanía Fernández-Gómez, Paloma García-Navas, Luis Javier Martínez-González, José Augusto Dávila-Fajardo, José Cabeza Barrera

**Affiliations:** 1 Department of Clinical Pharmacy, San Cecilio University Hospital, Institute for Biomedical Research, ibs.GRANADA, 18016 Granada, Spain; xandodv@gmail.com (X.D.-V.); estefania_fergo@hotmail.com (A.E.F.-G.); palomichi_96@hotmail.com (P.G.-N.); jose.cabeza.sspa@juntadeandalucia.es (J.C.B.); 2 Genomics Unit, Centro Pfizer-Universidad de Granada-Junta de Andalucía de Genómica e Investigación Oncológica (Genyo), 18016 Granada, Spain; albantunez@gmail.com (A.A.-R.); luisjavier.martinez@genyo.es (L.J.M.-G.); 3 English Department, Official Language School Leganes, 28915 Leganés, Madrid, Spain; josehood11@hotmail.com

**Keywords:** pharmacogenetics, clopidogrel, warfarin, acenocoumarol

## Abstract

There is a special interest in the implementation of pharmacogenetics in clinical practice, although there are some barriers that are preventing this integration. A large part of these pharmacogenetic tests are focused on drugs used in oncology and psychiatry fields and for antiviral drugs. However, the scientific evidence is also high for other drugs used in other medical areas, for example, in cardiology. In this article, we discuss the evidence and guidelines currently available on pharmacogenetics for clopidogrel, warfarin, acenocoumarol, and simvastatin and its implementation in daily clinical practice.

## 1. Introduction

In recent years, there have been important advances to understand how genetic variations are associated with the efficacy and/or toxicity of medicines. Some of these studies have been randomized clinical trials (RCT) for a variety of drug–gene combinations that have shown that the performance of pharmacogenetic tests before prescribing a medication can improve patient health outcomes [1,2,3,4,5]. For this reason, nowadays, there is an important need to generalize the clinical implementation of genomic medicine and pharmacogenetics (PGx).

There are some barriers that are preventing the integration of PGx in daily clinical practice [6]. Among them, we can highlight the lack of correlation between the different PGx guidelines and those published by other professional organizations (oncology, cardiology, etc.) [7], the lack of a clinically relevant PGx test panel, the need for training of health personnel and patients, and the lack of information on cost-effectiveness studies [8,9]. All of those reasons prevent physicians from demanding a proactive approach to PGx.

The most important PGx-based drug dosing guidelines are published by the Clinical Pharmacogenetics Implementation Consortium (CPIC), the Royal Dutch Association for the Advancement of Pharmacy—Pharmacogenetics Working Group (DPWG), and the Canadian Pharmacogenomics Network for Drug Safety (CPNDS). In general, there is enough agreement between the guidelines in terms of pharmacotherapeutic recommendations, but there are some aspects in which there are discrepancies due to the methodology used to support the dose recommendations that should be taken into account [10]. A large part of these PGx tests are focused on drugs used in oncology, antiviral drugs [11], and psychiatry fields. However, the scientific evidence is also high for other drugs used in other medical areas, for example, in cardiology. In our opinion, clinical pharmacists are ideal candidates to translate the PGx to clinical practice since they are qualified to lead efforts to guide optimal drug selection and drug dosing based on those results. However, most of them are not fully aware of the advance in the knowledge of PGx and some advanced pharmacist functions in applying clinical pharmacogenetic may require specialized education, training, or experience.

In this article we discuss the most relevant evidence currently available on PGx of cardiovascular drugs, focusing on those drugs with available PGx information and genetic tests, and its implementation into daily clinical practice. In order to provide a thorough analysis, we chose well-defined criteria. We decided to use the Pharmacogenomics Knowledgebase (PharmGKB) [12], where strength of evidence is rated in levels ranging from 1–4, with level 1 meeting the highest criteria. Thus, for this review, we focused on clopidogrel, warfarin, acenocoumarol, and simvastatin, as all of them are considered as level 1 for at least one variant (Table 1).

## 2. The Most Relevant Evidence in Pharmacogenetics of Drugs Used in Cardiology

### 2.1. Clopidogrel

Clopidogrel is a prodrug used as an antiplatelet in combination with acetylsalicylic acid in the treatment of acute coronary syndrome (ACS), including patients undergoing stent implantation after percutaneous coronary intervention (PCI) [13,14]. However, there is significant interpatient variability in the response of clopidogrel as a significant number of patients show high incidence of secondary cardiovascular events [15]. Several mechanisms were proposed for explaining the variable response to the drug, particularly when patients undergo PCI [16]. Subsequently, different genetic variants were associated with variability in response to the drug. The higher level of evidence is focused on the *CYP2C19* polymorphisms [17,18,19,20,21].

After absorption, 85% of the prodrug is inactivated by plasma esterases, and the remaining prodrug is activated in the liver by hepatic cytochrome isoenzymes. The conversion to its active metabolite depends partially on the CYP2C19 enzyme. Loss of function (LOF) *CYP2C19* alleles (*2 (rs424485), *3 (rs4986893), *4 (rs28399504), *5 (rs56337013), *6 (rs72552267), *7 (rs72558186), *8 (rs41291556), *9 (rs17884712), *10 (rs6413438), *22 (rs140278421), *24 (rs118203757), and *35 (rs12769205), mainly the CYP2C19*2 variant due to the higher frequency, were associated with lower levels of active clopidogrel metabolite, reduced platelet inhibition, and higher rates of cardiovascular events [22]. The variant CYP2C19*17 (rs12248560) is associated with a higher enzymatic activity, which means a lower on-treatment platelet reactivity [23,24,25,26] in response to clopidogrel when compared with homozygous wild-type carriers. However, CYP2C19*2/*17 carriers exhibited an increased platelet reactivity in response to clopidogrel, as compared with CYP2C19*1/*1 carriers [27], although data need to be consistently replicated.

For this reason, in 2010, The US Food and Drug Administration (FDA)-approved drug label for clopidogrel warned that tests are available to identify patients who were CYP2C19 poor metabolizers and suggested an alternative treatment in these patients, as they may have reduced effectiveness of the drug, therefore increasing the chance of secondary cardiovascular event rates in ACS and PCI patients, compared with patients with normal CYP2C19 function [28]. The European Medicines Agency (EMA) was positioned in a very similar way.

Nowadays, there have been some published clinical trials, meta-analyses, intervention studies, and many observational studies supporting the evidence of genotyping patients for clopidogrel use in ACS and PCI (Table 2 and Table 3).

#### 2.1.1. Large-Scale Studies in High-Risk Patients

Several large-scale studies in high-risk patients (PCI–stent) have evaluated the clinical implications of genetic variations in patients with coronary artery disease (CAD). In an article published at the beginning of 2009, Collet et al. [29] examined 259 patients <45 years with a first ACS with clopidogrel for at least one month, with 73% undergoing PCI. CYP2C19*2 carriers had a higher risk of death, ACS, and urgent revascularization (*p* = 0.0005, HR = 3.69) compared with non-carriers.

During this period, 2208 patients were enrolled in the French Registry of Acute ST-elevation and non-ST-elevation Myocardial Infarction (FAST-MI) [30], with 68.7% undergoing PCI. They evaluated whether some genes previously associated with altered pharmacokinetics of clopidogrel were also associated with cardiovascular events during the first year after ACS. Patients with two allelic variations *ABCB1* (TT) had a higher risk of cardiovascular events than those without allelic variation (CC) (Hazard ratio, HR = 1.72, 95%CI: 1.20–2.47). The risk of death, ACS, or stroke in patients with PCI was 3.58 times higher in patients who carry two copies *CYP2C19* LOF alleles compared to subjects without this allele.

In a Genome Wide Association Study (GWAS) performed in a large Amish population [32], it was seen that patients with increased age, greater BMI, higher triglycerides levels, and lower high-density lipoprotein cholesterol were associated with a poorer clopidogrel response; these variables explained less than 10% of the variation. In contrast, the heritability of ADP-stimulated platelet aggregation in response to clopidogrel was 73%, suggesting a substantial genetic component. They showed that in 277 ACS–PCI patients, the CYP2C19*2 polymorphism was associated with poorer cardiovascular outcomes (HR = 2.42, 95%CI: 1.18–4.99, *p* = 0.02).

##### Genetic Post-Hoc Substudies of the TRITON 38 Trial

The efficacies of clopidogrel and prasugrel were compared in the TRITON 38 trial [42] where 13,608 ACS–PCI–stent patients were included. Prasugrel reduced the percentage of cardiovascular death, ACS, or stroke at 15 months post-ACS, although it increased cases of bleeding.

Two genetic post-hoc studies of the TRITON-TIMI trial have been published by Mega et al. [17,22]. In a first approach [22], they evaluated the association between genetic polymorphisms in *CYP450* and secondary cardiovascular events in the clopidogrel subgroup. Patients who carried LOF alleles showed higher risk of cardiovascular death, ACS, or stroke compared with non-carriers (HR 1.53, 95%IC: 1.07–2.19, *p* = 0.01) and a higher risk of stent thrombosis (HR = 3.09, 95%CI: 1.19–8.00, *p* = 0.02). Months later, in a second article [17], they assessed the effect of the *ABCB1* polymorphism by itself and alongside variants in *CYP2C19* on cardiovascular outcomes. Both variants (CYP2C19*2 and *ABCB1*) were significant independent predictors of cardiovascular death, ACS, or stroke (*ABCB1* 3435 TT vs. CT/CC, HR 2.01, 95% CI: 1.30–3.11, *p* = 0.0017; *CYP2C19* LOF alleles carriers vs. non-carriers, HR 1.77, IC95%: 1.11–2.80, *p* = 0.0155). When the participants were divided into four groups on the basis of *ABCB1* 3435 C > T and *CYP2C19* status, those who did not carry at-risk genotypes in either gene had a significantly lower rate of cardiovascular death, ACS, or stroke at 15 months compared to those who were either carriers of *CYP2C19* LOF alleles, *ABCB1* 3435 TT homozygotes, or both (*p* = 0.0002).

During this period, another post-hoc study was published by Sorich et al. [31]. Individuals with a *CYP2C19* LOF genotype had a higher risk of cardiovascular death, ACS, or stroke than non-carriers in the clopidogrel group (RR 1.62, 95%CI: 1.27–2.06).

##### Genetics Post-Hoc Substudy of PLATO

In the PLATO trial [43], ticagrelor and clopidogrel were compared in patients with ACS, of whom only 64% had undergone PCI. In the genetic substudy including 10285 [33], PLATO demonstrated an increased rate of cardiovascular events in *CYP2C19* LOF carriers in the first 30 days of treatment with clopidogrel than in those with normal alleles, but they didn’t find significant difference in outcomes over the full follow-up period. Although *ABCB1* polymorphism was also genotyped, the combination of both variants (*CYP2C19* and *ABCB1*) comparing LOF carriers versus non-carriers in the clopidogrel group was not compared.

#### 2.1.2. Meta-Analyses of Large-Scale Studies

These studies and others were combined in several meta-analyses. In 2015, a systematic review of them was published by Osnabrugge et al. [44]. Most of the studies included in the meta-analysis showed statistical significance between polymorphisms and clinical endpoints, e.g., major adverse cardiovascular events (MACE) and stent thrombosis. However, the meta-analysis concluded that the association between *CYP2C19* LOF alleles and clinical efficacy of clopidogrel differed widely with regard to assessment, interpretation of high heterogeneity, and publication bias. Also, personalizing antiplatelet management based on genotyping is not supported by the currently available evidence [44].

#### 2.1.3. Non-Randomized Clinical Trials

Others prospective, non-RCTs of *CYP2C19* genotype-guided clopidogrel therapy with clinical outcomes have been performed. In 2016, our group published a study with the aim of analyzing if the *CYP2C19/ABCB1* genotype-guided approach, in which the choice of antiplatelet therapy is based on the genetic test, could reduce the rates of cardiovascular events and bleeding compared to a non-tailored approach in 719 patients (more than 86% with ACS) who had undergone PCI with stent [35,45]. The primary endpoint (composite of cardiovascular death, ACS, or stroke during 12 months after intervention) occurred in 10.1% in the genotyping group and in 14.1% in the control group (HR 0.63, 95% CI (0.41–0.97), *p* = 0.037). The results showed that there was no difference in major and minor bleeding between the two groups (4.1% vs. 4.7%, HR = 0.80, 95%CI (0.39–1.63), *p* = 0.55) [35].

#### 2.1.4. Clinical Trials

In 2016, Shen et al. published a study where 628 CAD patients undergoing PCI were divided into a control group (n = 319) and an intervention group (*n* = 309), which were tested for *CYP2C19* [36]. In the intervention group, extensive metabolizer patients received 75 mg daily of clopidogrel, intermediate metabolizer patients received 150 mg daily of clopidogrel, and poor metabolizer patients received ticagrelor 90 mg twice daily. The control group received clopidogrel 75 mg daily. The rates of MACE in the intervention group were lower than those in the control group at 1, 6, and 12 months (*P* = 0.010). There were no differences in the rates of bleeding between both groups (*P* > 0.05).

In 2012, a clinical trial testing this strategy was published (RAPID GENE Study, NCT01184300) using a novel point-of-care genetic test to identify carriers of the CYP2C19*2 allele, which aimed to assess a pharmacogenetic approach to dual antiplatelet treatment after PCI [37]. The CYP2C19*2-pharmacogenetic strategy after PCI was effective in reducing high on-treatment platelet reactivity at day 7 in CYP2C19*2 carriers. Recently, the same group confirmed that the identification of these genetic variants in patients with STEMI receiving PCI is feasible at the bedside and demonstrated that treatment of CYP2C19*2, *17, and *ABCB1* TT carriers with prasugrel resulted in a significant reduction in high platelet reactivity after 1 month compared to an augmented dosing of clopidogrel [38].

In 2013, 600 patients with CAD undergoing PCI randomly received a personalized antiplatelet therapy or conventional antiplatelet treatment and followed for the 180-day period after randomization. In the intervention group, the antiplatelet therapy was chosen according to CYP2C19 phenotype. In the control group, the patients received conventional antiplatelet treatment. The incidence of the primary end point (MACE) was 9.03% for patients assigned to conventional treatment and 2.66% for patients assigned to personalized therapy (*p* < 0.01), without differences in bleeding events between the 2 groups [39].

The PHARMCLO RCT is another clopidogrel pharmacogenetic study published in 2018 [40]. It is a prospective, multicenter RCT achieved in Italy between 2013 and 2015. 888 patients hospitalized for ACS were randomly assigned to standard of care or the PGx intervention arm, which included the genotyping of *ABCB1*, CYP2C19*2, and CYP2C19*17 using an ST Q3 system that provides data within 70 min at each patient’s bedside. The patients were followed up for 12 months for the primary composite endpoint of cardiovascular death and the first occurrence of nonfatal myocardial infarction, nonfatal stroke, and major bleeding was defined according to Bleeding Academic Research Consortium type 3 to 5 criteria. The study was prematurely stopped at only 25% of prespecified enrollment, because of the lack of in vitro diagnosis certification of the genotyping instrument. However, despite only enrolling a fraction of the anticipated sample size, the primary endpoint occurred in 15.9% in the intervention arm and in 25.9% in the standard-of-care arm (HR: 0.58; 95% CI: 0.43 to 0.78; *p* < 0.001).

In the Netherlands, a multicenter trial named Cost-effectiveness of Genotype Guided Treatment With Antiplatelet Drugs in STEMI Patients: Optimization of Treatment (POPular Genetics, NCT01761786) started in 2011 to assess the efficacy, safety, and cost-effectiveness of the *CYP2C19* genotype-guided antiplatelet treatment strategy, using clopidogrel in non-carriers of the CYP2C19*2 or *3 allele and ticagrelor or prasugrel in carriers of the CYP2C19*2 or *3 allele in 2500 STEMI patients. [41]. Similarly, the Tailored Antiplatelet Therapy Following PCI (TAILOR-PCI) is a multi-site, open label, prospective, randomized trial, where 5000 patients with ACS or stable CAD who underwent PCI with stent will be recruited and randomized to receive a conventional therapy or a *CYP2C19* genotype-based anti-platelet therapy approach (NCT01742117).

#### 2.1.5. Meta-Analyses

In 2018, a meta-analysis performed by Kheiri et al. [46] was published, including six RCTs with a total of 2371 patients. Of those studies, only three trials included ACS patients [39,40,47], Tuteja et al. [48] was not published and included CAD patients, Tomaniak et al. [49] included stable CAD patients, and Robert et al. [37] mainly included CAD (only 37% ACS). The results showed that the rate of MACEs was not significantly different between intervention groups and control groups (8.9% vs. 12.8%, RR0.67 IC 0.35–1.27, *p* = 0.22, I^2^ = 74%). The high heterogeneity was due to inconsistency in definitions of MACE among the trials, different follow-up times, different genotype testing systems with varied tested alleles, and a variety of dosing algorithms. Sensitivity analysis by excluding the unpublished trial [48] resulted in a significant reduction of MACE in favor to the genotype-guided group with almost no heterogeneity (RR 0.55, 95%CI 0.41–0.74, *p* < 0.01, I2 = 2%). Similarly, a sensitive analysis by including only the three trials that assessed genotype testing exclusively in ACS patients suggested a significant reduction of MACE.

#### 2.1.6. Guidelines

Taking into account all the commented information, clopidogrel should be considered as an ideal target for pharmacogenetic intervention, at least in high-risk patients, due to the high level of evidence associated with the reduction of cardiovascular events rates and because there are other alternatives of antiplatelet drugs which are not affected by *CYP2C19* polymorphisms. This is supported by CIPC and DPGW guidelines, which have labelled the clopidogrel–CYP2C19 interaction as 1A. The CPIC and the DPWG recommend the use of genetic information to guide clopidogrel therapy, especially in ACS patients who have undergone PCI [50,51,52,53]. Both guidelines recommend considering an alternative drug for CYP2C19 poor or intermediate metabolizers due to increased risk for reduced response to clopidogrel.

In low-risk patients (no PCI–stent), no relationship was found between CYP2C19*2 status and adverse outcomes [34,54]. Despite the evidence and the PGx guidelines, the current guidelines for the treatment of ACS by the American Heart Association/American College of Cardiology (AHA/ACC) and the guideline recommendations by European Society of Cardiology do not make references to the possibility of carrying out the pharmacogenetic test even in high-risk patients (ACS–PCI–stent) and, in consequence, they contradict the CPIC and DPGW guidelines and the FDA and EMA recommendations. A recent and very good review of the lack of updating of the American and European cardiology guidelines of ACS with respect to the clopidogrel test has been published by Luzum and Cheung [7]. The AHA/ACC recommends against routine pharmacogenetic testing for clopidogrel because no RCTs have demonstrated the testing improves patient’s outcomes [55]. According to the authors of this article [7], the level of evidence supporting by *CYP2C19* genotype-guided clopidogrel therapy in patients that received PCI is at least as strong as the other genetic tests recommended by the AHA/ACC. Fortunately, several institutions have implemented pharmacogenetic testing for clopidogrel despite the negative recommendation by AHA/ACC [35,45,56,57] and they found improvement in the clinical results of patients [58,59].

#### 2.1.7. Cost-Effectiveness Studies

There are different cost-effectiveness studies supporting that preventive genotyping test of *CYP2C19* is cost-effective and could be applicable in clinical practice [60,61,62,63]. In collaboration with The Golden Helix Foundation, in 2018 we performed a cost-effectiveness analysis of pharmacogenomic-guided antiplatelet treatment using the data published [60] by our team regarding Spanish ACS patients who underwent PCI [35]. This study is one of the very few that aims to compare the cost-effectiveness of antiplatelet treatment modalities retrospectively versus prospectively genotyped patients for the CYP2C19*2, CYP2C19*3, and CYP2C19*17 alleles. Our analysis suggests that the prospective treatment strategy costs slightly less and has a marginally higher effectiveness compared to the retrospective group.

In 2015, Johnson et al. [64] estimated the financial impact of *CYP2C19* genotyping in a theoretical cohort of 1000 patients with ACS who received PCI–stent implantation and were treated with clopidogrel, prasugrel, or ticagrelor in a managed care setting. The budget-impact analysis used published event rates from primary literature to estimate costs of events analysis for three different scenarios: Scenario A, no *CYP2C19* genotyping; Scenario B, 50% of patients received *CYP2C19* genotyping with appropriate treatment based on genotype; and Scenario C, 100% of patients received *CYP2C19* genotyping with appropriate treatment based on genotype. They concluded that important financial benefits may be realized through use of genotype-guided antiplatelet therapy to reserve prasugrel or ticagrelor use for patients with reduced CYP2C19 activity to avoid costs associated with adverse cardiac events.

A systematic review of economic evaluation of pharmacogenetic testing for prevention of adverse drug reactions was published in 2016 [65]. There was evidence supporting the cost effectiveness of testing for different drugs, including clopidogrel.

### 2.2. Warfarin

Warfarin is an anticoagulant widely used for the prevention of thromboembolic and hemorrhagic episodes [66,67]. The drug decreases the activation of vitamin K-dependent coagulation factors by inhibiting the enzyme epoxide reductase [68]. Due to the great variability in the individual response and a narrow therapeutic window, there is a significant risk of thromboembolism if the doses are less than adequate, or of hemorrhage in case of overdose [69] for patients with the same International Normalized Ratio (INR) target [67].

Different algorithms based on clinical parameters such as age, weight, and height were published, but these are inaccurate and explain only 12–22% of the dose variation [70]. In recent years, different algorithms also including genetic polymorphisms that affect enzymes that mediate the metabolism of the drug were published. The most popular variants are the genes that encode CYP2C9 and the epoxide reductase of vitamin K in the *VKORC1* gene, which affect the properties pharmacokinetics and pharmacodynamics of warfarin [71].

The *CYP2C9* gene has many allelic variants. Individuals homozygous for the wild-type allele (CYP2C9*1) have a “normal metabolism” of S-warfarin, the most potent form of this drug. The *2 (rs1799853) and *3 (rs1057910) alleles have a reduced enzymatic activity for the excretion of the drug, which entails a decrease of around 30% and 80%, respectively [67].

On the other hand, the enzyme encoded by *VKORC1* catalyzes the reduction of vitamin K, a necessary step to activate the coagulation factors. Its polymorphism, -1639G > A (rs9923231), is associated with an increased sensitivity to warfarin and the decrease in the amount required [67].

#### 2.2.1. Genotype-Guided Algorithms

Gage et al. [72] published the first warfarin genotype-guided algorithm, including 369 patients who had a stable dose of warfarin. As genetic variants they only included *CYP2C*9*2 and *3 and as demographic and clinical variants they included age, body surface area, gender race, target INR, and comedication (amiodarone and simvastatin). The algorithm presented a coefficient of determination (R^2^) of 39%. In 2008, the same group published a new algorithm [70] which improved the R^2^ = 54%. They increased the number of patients to 1015 and included another genetic variant, *VKORC1* 1639G>A, smoking status, and the indication of the drug [70].

For these reasons, in 2007, the FDA approved the inclusion in the drug’s data sheet of a dosage table that recommends taking into account polymorphisms -1639G>A in the *VKORC1* gene and *2 and *3 in the *CYP2C9* gene, to set the initial dose [73]. The decision was made because many studies showed that these polymorphisms were associated with great variability in the response to warfarin.

Other pharmacogenetic algorithms have been proposed that take into account both the genetic polymorphisms and the clinical variables of the patients to estimate the maintenance dose of warfarin [70,74,75,76], which seem to have greater accuracy than the tables in the technical data sheet [77]. However, they do not evaluate whether the pharmacogenetic algorithm can lead to the improvement of clinical results, such as % INR out of range, time to reach the INR, and frequency of appearance of thrombotic or hemorrhagic events. These algorithms explain about 50% of the dose variation [70,77], with greater benefit at the end of the dosage [74].

#### 2.2.2. Clinical Trials

Different clinical trials have been published in recent years, measuring in these cases clinical outcomes such as thrombotic and hemorrhagic adverse events, time to reach the INR, and time in therapeutic range (Table 4). Among all of them we would like to highlight two pharmacogenetic trials of warfarin therapy, European Pharmacogenetics of Anticoagulant Therapy (EU-PACT), and Clarification of Optimal Anticoagulation through Genetics (COAG), reported by Pirmohamed et al. [3] and Kimmel et al. [78], respectively, that were published at the same time with contradictory messages. Pirmohamed et al. [3] recruited 455 patients with atrial fibrillation (AF) or venous thromboembolism. For patients assigned to the genotype-guided group, warfarin doses were prescribed according to a PGx algorithm for the first five days. Patients in the control group received a 3-day loading-dose regime (fixed-dose strategy). After the initiation period, the treatment of all patients was managed according to routine clinical practice. The primary outcome was the percentage of time in the therapeutic range (TTR) of 2.0 to 3.0 for the INR during the first 12 weeks after warfarin initiation. The mean percentage of time in the therapeutic range was 67.4% in the genotype group as compared with 60.3% in the control group (*p* < 0.001). The mean time to reach a therapeutic INR was 21 days in the genotype group as compared with 29 days in the control group (*p* < 0.001).

Kimmel et al. [78] recruited 1015 patients, 80% with AF or deep-vein thrombosis or pulmonary embolism. The dose of warfarin during the first five days of therapy was determined according to a dosing algorithm that included both clinical variables and genotype data, or to one that included clinical variables only. The primary outcome was the percentage of TTR from day 4 or 5 through day 28 of therapy. At 4 weeks, the mean percentage of TTR was 45.2% in the genotype group and 45% in the control group (*p* = 0.91). In North Africans patients, the mean percentage of time in the therapeutic range was less in the genotype group than in the control group.

One possible explanation could be the difference observed in the genotyping results [79], as the prevalence of homozygotes, who required the most significant dosing changes, was 17% in EU-PACT versus 11% in the COAG trial for the *VKORC1* variant and 3.4% in EU-PACT versus 1% in the COAG trial for the CYP2C9*2 and CYP2C9*3 variants. According to Shaw et al. [80] some key differences between the two studies were length of follow-up time (12 weeks for EU-PACT and 4 weeks for COAG), determination of dose in the non-genotype group (fixed dose in EUPACT and clinical dosing algorithm in COAG), patient ancestry (2% non-European in EU-PACT and 33% non-European in COAG), and the availability of genetic test results (EU-PACT genotype results were available in approximately 2 h, COAG genotype results were not available before the first dose for 55% of patients). Another possible explanation could be the different diseases affecting the patients which could also affect the warfarin dosing.

After these trials, others have been published in which it was demonstrated that the pharmacogenetic algorithm could reduce the time to reach the maintenance dose [81] or that a lower number of thrombotic or hemorrhagic events was achieved [82]. In 2017, the GIFT randomized clinical trial was published [82] to determine if genotype-guided dosing improves the safety of warfarin initiation among undergoing hip or knee arthroplasty. The results showed that genotype-guided warfarin dosing, compared with clinically guide dosing, reduced the combined risk of major bleeding, INR of 4 or greater, venous thromboembolism, and death. Further research is necessary to determine the cost-effectiveness of personalized warfarin dose.

#### 2.2.3. Meta-Analyses

The efficacy of the different algorithms published has been analyzed in different meta-analyses. In 2014, Goulding et al. [83] performed a meta-analysis including nine RCTs which evaluated genotype-guided warfarin dosing. Analysis of the percentage of TTR showed a statistically significant benefit in favor of genotype-guided warfarin dosing (mean difference = 6.67; 95% CI 1.34, 12.0, I^2^ = 80%). Similarly, they found a statistically significant reduction in minor bleeding, major bleeding, and thromboembolism associated with genotype-guided warfarin dosing, RR 0.57 (95% CI 0.33, 0.99; I^2^ = 60%). As conclusion, they considered that the genotype-guided warfarin dosing algorithm could improve the clinical effectiveness.

In 2015, Liao et al. [84] performed a meta-analysis including seven trials. In total, 1910 patients were included, 960 patients who received genotype plus clinical algorithm of warfarin dosing and 950 patients who received clinical algorithm only. The results showed that the percentage of TTR in the genotype-guided group improved compared with the standard group in the RCTs when the initial standard dose was fixed (95% CI 0.09–0.40; I^2^ = 47.8%), but not when the studies were using no fixed initial doses. They did not find any difference in the incidences of adverse events (RR 0.94, 95% CI 0.84–1.04; I^2^ = 0%, *p* = 0.647) and death rates (RR 1.36, 95% CI 0.46–4.05; I2 = 10.4%, *p* = 0.328) between the two groups. 

In 2014, Tang et al. [85] performed a systematic review and meta-analysis including ten studies with a total of 5299 patients. The control groups were treated with fixed dose or clinical algorithms. The results showed that patients in the genotype-guided group had higher percentage of TTR than the control group (I^2^ = 84%) and reduced risk for hemorrhagic complications (I^2^ = 0%).

In 2015, Belley-Cote et al. [86] performed a systematic review and a meta-analysis including 12 studies (3217 patients) (11 studies with warfarin and 1 study with acenocoumarol and phenprocoumon). The control group was treated with fixed dose or clinical algorithms. They concluded that the genotype-guide approach compared to the non-genotype guide was not found to decrease a composite of death, thromboembolism, and major bleeding (I^2^ = 10%), but the results improved the TTR (I^2^ = 79%) in comparison with fixed vitamin K-antagonist dosing, but not with the clinical algorithms.

In 2015, Li et al. [87] conducted a meta-analysis of the published RCTs comparing PGx algorithm-based warfarin dosing with clinical variants or standard protocols (control group). A total of ten RCTs were retrieved for the meta-analysis, including 2601 participants. No heterogeneity was found for the primary or subgroup analyses of major bleeding and thromboembolic events (I^2^ ≤ 25%). The results showed that major bleeding and thromboembolic events were significantly lower in the PGx group than in the control group. Similarly, there was a trend towards increased percentage of TTR (*p* = 0.05) in the PGx group, but no difference was observed for over-anticoagulation (INR > 4).

In 2015, Dahal et al. [88] performed a meta-analysis including ten RCTs, which included 2505 patients, and compared PGx algorithm-based warfarin dosing with clinical variants or standard protocols (control group). After one month, improved percentage of TTR and major bleeding incidence (I^2^ = 26%) was observed, making this a cost-effective strategy in patients requiring longer anticoagulation therapy.

In 2015, Shi et al. [89] included 11 trials involving 2678 patients in a meta-analysis. The results showed that the PGx approach did not improve the TTR compared to control group (I^2^ = 82%), although it significantly shortened the time to maintenance dose and the time to first therapeutic INR. Moreover, the PGx approach significantly reduced the risk of adverse events and major bleeding (I^2^ = 15%).

#### 2.2.4. Guidelines

In 2017 an update of the CPIC guidelines for pharmacogenetics-guided warfarin dosing was published [90]. Evidence from the literature has permitted including another variant (*CYP4F2* rs12777823), related to the limitation of the excessive accumulation of vitamin K that improves the accuracy of dose prediction [91]. Similarly, the CPNDS clinical recommendation group has published guidelines for the use of pharmacogenetic testing for variants in *VKORC1* and *CYP2C9* in adult and pediatric patients with an indication for warfarin [80]. They recommend testing for the *VKORC1* SNP (Single nucleotide polymorphism) -1639G>A (rs9923231) and the *CYP2C9* alleles *2 and *3 in order to better guide warfarin dosage.

#### 2.2.5. Cost-Effectiveness Studies

Similar to clopidogrel, there are different cost-effectiveness studies supporting that preventive genotype testing for warfarin is cost-effective. Most of the studies have demonstrated that the genotype-guided dosing approach can lead to reduced bleeding and improve quality-adjusted life-years (QALYs) gained. In 2009, Eckman et al. [92] examined the cost-effectiveness of genotype-guided dosing (CYP2C9*2, CYP2C9*3, and/or *VKORC1*) versus standard induction of warfarin therapy for patients with nonvalvular AF using the Markov decision model. Effectiveness was measured in QALYs. They concluded that warfarin-related genotyping is unlikely to be cost-effective for typical patients with nonvalvular AF, but may be cost-effective in patients at high risk for hemorrhage who are starting warfarin therapy.

In 2009, Leey et al. [93] evaluated the potential clinical and economic outcomes of genotype-guided warfarin therapy in elderly patients newly diagnosed with AF. A decision tree was designed to represent the medical decision (pharmacogenetic testing or not) and the main clinical outcomes (embolic stroke, bleeding). They found that any reduction in major bleeding as a result of pharmacogenetic testing would lead to improved utility.

In 2013, Pink et al. [94] compared the cost-effectiveness of a variety of clinical dosing algorithms, pharmacogenetic dosing algorithms, and new anticoagulant-based therapies. Warfarin pharmacogenetic algorithms were more cost-effective than clinical-based dosing algorithms. Neither dabigatran nor rivaroxaban were cost-effective options, but apixaban appeared to be the most cost-effective treatment when warfarin therapy was poorly controlled.

### 2.3. Acenocumarol

Acenocoumarol is a vitamin K epoxide reductase inhibitor. The drug inhibits recycling of the inactive oxidized to the active reduced form of vitamin K. It is used for the prevention of thromboembolic and hemorrhagic episodes [95]. As with warfarin, different polymorphisms in *CYP2C9* and *VKORC1* genes have been associated with the efficacy of the drug.

#### 2.3.1. Pharmacogenetics Algorithms

In the last years, several pharmacogenetics algorithms have also been published for acenocoumarol in diverse populations. Verde et al. constructed an ‘‘acenocoumarol-dose genotype score’’ based on the number of alleles associated with a higher acenocoumarol dosage carried by each participant for each polymorphism [96]. They concluded that this approach could discriminate patients requiring high acenocoumarol doses to achieve the target. Rathore et al. [97] and Krishna et al. [98] published two algorithms for Indian populations, including demographic, clinical, and genetic variants, and the coefficients of determinations obtained were 41% and 61.5%, respectively [97,98]. Four algorithms were developed for European populations. In 2011, the European Pharmacogenetics of Anticoagulant Therapy (EU-PACT) study group published an algorithm including *CYP2C9* and *VKORC1* variants and clinical variables (age, sex, weight, height, and amiodarone use). The PGx algorithm explained 52.6% of the dosage variance, whereas the non-genotype algorithm explained 23.7% [99]. Borobia et al. [100] developed an algorithm for a cohort of 147 patients with thromboembolic venous disease who were on stable doses including clinical variables (age, body mass index (BMI), amiodarone use, and enzyme-inducer use) and genetic variations of *CYP2C9*, *VKORC1*, *CYP4F2,* and *APOE*. The clinical factors explained 22% of the dose variability, which increased to 60.6% when pharmacogenetic information was included (*p* < 0.001) [100]. Cerezo-Manchado et al. [101] published an algorithm including 973 patients undergoing anticoagulation therapy. The algorithm was composite of clinical factors (age and BMI) and genetic variants (*VKORC1*, *CYP2C9,* and *CYP4F2* variants). The algorithm explained 50% of the variance in the acenocoumarol dosage, whereas the clinical algorithm explained 16% [101].

In 2016, a new algorithm including clinical (age, weight, amiodarone use, enzyme inducer status, international normalized ratio target range) and genetic variables (CYP2C9*2 (rs1799853), CYP2C9*3 (rs1057910), *VKORC1* (rs9923231), and *CYP4F2* (rs2108622)) to predict the most appropriate acenocoumarol dosage for stable anticoagulation in a cohort of 685 Spanish patients was published by our team in collaboration with Hospital de la Paz [102]. The R^2^ explained by the algorithm was 52.8% in the generation cohort and 64% in the validation cohort. When the patients were classified into three dosage groups according to the stable dosage (<11 mg/week, 11–21 mg/week, >21 mg/week), the percentage of correctly classified patients was higher in the intermediate group, whereas differences between pharmacogenetic and clinical algorithms increased in the extreme dosage groups.

The utility of PGx-guided acenocoumarol and phenprocoumon prescribing during therapy initiation was investigated in a prospective trial: EU-PACT [2]. The genotype-guided dosing algorithm included clinical variables and genotyping for *CYP2C9* and *VKORC1* and the control-dosing algorithm included only clinical variables for the initiation of acenocoumarol or phenprocoumon treatment in patients with AF or venous thromboembolism. The primary outcome was the percentage of TTR in the 12-week period after the initiation of therapy. The intervention arm showed no statistically significant difference in the mean percentage of time in the therapeutic INR range compared with the control group. (*p* = 0.52). Some years later, to explore the potential reasons for these findings, the same team performed subanalyses stratifying the data by the *VKORC1* and *CYP2C9* genotypes [103]. They realized that the EU-PACT genetic-guided dose initiation algorithms for acenocoumarol and phenprocumon could have predicted the dose overcautiously in the *VKORC1* AA-CYP2C9*1/*1 subgroup.

#### 2.3.2. Meta-Analyses

Recently, a meta-analysis has been published [104] including 15,754 patients. The CYP4F2*3 polymorphism was consistently associated with an increase in mean coumarin dose (+9% (95% CI 7–10%), with a larger effect in females, in patients taking acenocoumarol, and in Europeans. The inclusion of the CYP4F2*3 in dosing algorithms slightly improved the prediction of stable coumarin dose. New pharmacogenetic equations potentially useful for clinical practice were derived [104].

#### 2.3.3. Guidelines

The DPWG recommends checking INR more frequently after initiating or discontinuing NSAIDs in individuals taking acenocoumarol with at least one CYP2C9*2 or *3 allele. While *VKORC1* genotype has been found to contribute to acenocoumarol dose variability, there are no dosing recommendations at this time because of strict INR monitoring by the Dutch Thrombosis Service. They recommend checking INR more frequently in patients with the AA genotype [52,53].

### 2.4. Simvastatin

Simvastatin is a lipid-modifying agent used in the treatment of different kinds of hypercholesterolemia, one of the most significant risk factors in cardiovascular disease. Furthermore, it has shown a decrease of morbimortality in atherosclerotic cardiovascular disease patients, even those with normal cholesterol levels.

Simvastatin has been significantly associated with skeletal muscle toxicity (myalgia, myopathy, and rhabdomyolysis) [105], especially a high risk of myopathy with a dose of 80 mg daily [106].

Simvastatin inhibits the cholesterol production by competitive inhibition of HMG-CoA reductase, increasing the number of LDL receptors on liver cells. Its metabolism is mediated by many CYP isoenzymes (CYP3A4, CYP3A5, CYP2C9, CYP2C19, etc.) and its movement depends on SLCO1B1 and ABCB isoforms [12]. The FDA approved a drug label for simvastatin indicating that simvastatin is a substrate for the transport protein SLCO1B1. Genetic variants contained in genes encoding these transporters and metabolic enzymes expression may affect a patient’s simvastatin response.

#### 2.4.1. Observational Studies

The SLCO1B1 enzyme, encoded by the *SLCO1B1* gene, is involved in the simvastatin carriage from intestinal to liver cells [12]. In this gene, there may be a variant, the *c.521T>C* (rs4149056), considered in SLCO1B1*5, *15, and *17 haplotypes, which is the only one which has reached the highest level of evidence about its association with interindividual differences in simvastatin patients’ responses [107].

Among healthy individuals, the *SLCO1B1 521 CC* genotype has been associated with higher plasma concentration of simvastatin [108,109]. This genotype has been also significantly related to an increased likelihood of muscular disease in patients treated with simvastatin after ACS (OR = 16.9; 95%CI = 4.7–61.1; *p* = 6.0E-4) and with a higher risk of muscular disease in occlusive vascular disease or diabetes patients (RR = 2.6; 95%CI = 1.3–5; *p* = 0.004) [110] when compared with *CT* or *TT* genotypes. Furthermore, *CC* compared to *CT* genotype confirmed these results, with SLCO1B1*5 CC individuals showing a higher risk of muscular disease.

#### 2.4.2. Clinical Trials

The STRENGTH study was a pharmacogenetics study of statin efficacy and safety. 509 patients were randomized to atorvastatin, simvastatin, or pravastatin. The composite adverse event (discontinuation for any side effect, myalgia, or CK > 3× baseline during follow-up) occurred in 99 subjects. SLCO1B1*5 genotype and female sex were associated with mild statin-induced side effects. In patients with hypercholesterolemia, the *C* allele was associated with increased risk of adverse drug events when treated with atorvastatin, pravastatin, or simvastatin (OR = 1.7; 95%CI = 1.04–2.08; *p* = 0.03) [111]. Furthermore, regarding patients with hyperlipidemia treated with simvastatin only, carrying the SLCO1B1*5 (*CC/CT* vs. *TT*) allele was associated with higher risk of muscular diseases [112].

#### 2.4.3. Guidelines

The FDA recommends against 80 mg daily simvastatin dosage. In patients with the *C* allele at *SLCO1B1* rs4149056, there are modest increases in myopathy risk even at lower simvastatin doses (40 mg daily); if optimal efficacy is not achieved with a lower dose, alternate agents should be considered. This annotation is based on the CPIC guideline for simvastatin and *SLCO1B1* [107].

## 3. Discussion

There are many barriers that hinder the implementation of PGx in daily clinical practice. In our opinion, once the regulatory agencies recommend doing the pharmacogenetic test, their implementation should not be delayed. However, as we have shown in this review, it is not performing at the expected rate as there is a disconnection between drug labels and the standard of care in daily clinical practice.

Regulators are often confronted with challenges involved in translating data from pharmacogenomic studies into clinically relevant and meaningful product information, starting with the level of scientific evidence required to justify the inclusion of PGx data in the product information [11]. In case of the new drugs, there are two guidelines on pharmacogenomics during drug development and the post-authorization phase, respectively [113].

With them, the EMA intends further to enable the potential of PGx during drug development and surveillance and to gain insight into the associated scientific challenges and discuss potential solutions. The guidelines are expected to improve genomic data-informed drug development and clinical experience, thereby promoting understanding of interindividual drug response variations and, consequently, providing guidance towards more personalized treatments in the interest of patients and the public.

However, older drugs, such as warfarin, acenocoumarol, simvastatin, and clopidogrel, have been subject to pharmacogenomic scrutiny by the EMA after their authorization [11]. So, the implementation of the PGx test should be easy.

Knowing the evidence commented above for clopidogrel, it would appear therefore that genotype-directed therapy with clopidogrel would more likely benefit a population with the greatest risk (PCI–stent). Administration of stronger antiplatelet drugs in low-risk patients would probably not reduce the thrombotic risk, but would increase the risk of bleedings [114]. However, prescribing stronger antiplatelet drugs only to the high-risk patients resistant to clopidogrel could add to a new era of personalized medicine. The old “one size fits all” regime should come to an end; tailored antiplatelet therapy is taking over, based on the patient’s individual risk factors for atherothrombotic events such as HPR (High platelet reactivity), diabetes, ACS, and genetic polymorphisms.

The AHA/ACC guidelines recommend against routine pharmacogenetic testing because there are no clinical trials published yet [14,55], but, before the no recommendation to do the PGx test of clopidogrel by the AHA/ACC due to the lack of RCT, there was already one published by Xie et al. [39], in which it was shown that the incidence of secondary cardiovascular events was lower in the intervention group with respect to the control group, as we have commented in this review. It is probably necessary to wait for the clinical results of the rest of RCTs discussed in this review and wait for the next update of the AHA/ACC.

In our opinion, the level of evidence supporting *CYP2C19* genotype-guided clopidogrel therapy in patients undergoing PCI is high enough and endorsed by the regulatory agencies for the AHA/ACC guide to include it, given that the AHA/ACC recommends other PGx tests in absence of prospective clinical trials. The evidence showed by large-scale observational studies in high-risk patients suggests that the *CYP2C19* LOF alleles are associated with MACE. The meta-analysis published by Osnabrugge et al. [44] showed that there is high heterogeneity between studies and publication bias and since the validity of the overall conclusion of a meta-analysis depends, to a large extent, on the homogeneity of the studies included, in our opinion this should be considered as an important limitation of the meta-analysis [115]. Regarding clinical trials, one non-RCT [35] and five RCTs [36,37,39,40,41] (and TAILOR-PCI) show that PGx tests can improve results in the health of patients. Similarly, the meta-analysis including these RCTs [46] showed high heterogeneity due to inconsistency in definitions of MACE, different follow-up time, different genotype systems, etc. Despite this, when an unpublished article [48] was excluded from the meta-analysis, there was almost no heterogeneity and the results showed that the intervention group reduced MACE and was statistically significant. Moreover, several studies have shown that the application of PGx to clopidogrel is cost-effective.

Regarding warfarin, several PGx algorithms, including clinical variables, have been published and considered efficient methods for determining individual stable warfarin dose. Although there is enough information to show the association between genetic variants and warfarin dose, the results published in RCTs are controversial, mainly because of the complexity, the important differences in the design, the differences observed in the prevalence of the genotyping results [79], and the different diseases included, all of which could affect warfarin dosing. In spite of this, most of the meta-analyses of RCTs show at least an improvement of percentage of TTR when using the pharmacogenetic algorithm compared with the standard protocol.

Sample size in genotyping trials (e.g., Tailored Antiplatelet Initiation to Lessen Outcomes Due to Decreased Clopidogrel Response after Percutaneous Coronary Intervention [TAILOR-PCI trial, ClinicalTrials.gov number, NCT01742117) should probably be calculated on the basis of the prevalence of reduced-function or loss-of-function alleles that affect the phenotype, since we do not anticipate a difference in outcomes in patients without such mutations [116].

In our opinion, the greatest benefit of the implementation of the PGx test for warfarin would be in those patients who initiate the treatment; then we could anticipate if the drug is going to work or if it is better to prescribe a new oral anticoagulant drug and in those patients who, after a reasonable period, do not manage to maintain the INR in order to justify that it is due to poor metabolism of the drug.

Observational evidence suggests that the use of a genotype-guided dosing algorithm may increase the effectiveness and safety of acenocoumarol therapy. Although it’s important to note that the published algorithms differ in the kind of patients and diseases, the clinical and genetic included variables, and the methods used to develop the predictive models.

However, the only clinical trial achieved reported initially that genotype-guided dosing of acenocoumarol (and phenprocoumon) did not improve the percentage of time in the therapeutic INR range during the 12 weeks after the initiation of therapy. After performing subanalyses stratifying the data by the *VKORC1* and *CYP2C9* genotypes [103], they realized that the EU-PACT genetic-guided dose initiation algorithms for acenocoumarol (and phenprocumon) could have predicted the dose overcautiously in the *VKORC1* AA-CYP2C9*1/*1 subgroup.

This trial had limitations that could have influenced the final result; on the one hand, it is important to know that the CYP2C9 enzyme has much less influence on the pharmacokinetics of phenprocoumon than on the pharmacokinetics of warfarin [117], but on the other hand, the number of patients included was lower than the number required according to the power calculation.

In our opinion, those patients who fail to reach or maintain the INR after a period of treatment could benefit from the PGx test. More studies are necessary to implement the PGx test before the prescription for guiding the dose of acenocoumarol.

Regarding simvastatin, an RCT showed that SLCO1B1*5 genotype and female sex were associated mild statin-induced side effects. As the FDA and CPIC guidelines recommend the PGx test, this should at least be used when symptomatology of myopathy starts.

In conclusion, PGx tests for clopidogrel in high-risk patients and warfarin in patients including all indications could begin to be implemented in daily clinical practice, similar to simvastatin tests. Acenocoumarol should be limited to patients who do not reach the INR after a certain time of treatment. The algorithm could improve acenocoumarol dosage selection for patients who will begin treatment with this drug, especially in extreme-dosage patients. Further studies are necessary to confirm that the PGx test for acenocoumarol is ready for use.

## Figures and Tables

**Table 1:** Drug-genes interactions reported in this article and the corresponding PGx guidelines and the level of evidence. CPIC: Clinical Pharmacogenetics Implementation Consortium, DPWG: Dutch Pharmacogenetics Working Group, CPNDS: Canadian Pharmacogenomics Network for Drug Safety.

Drugs	Genes	PGx Guidelines	Level of Evidence
Clopidogrel	*CYP2C19*	CPIC, DPWG	1A
Warfarin	*CYP2C9*, *VKORC1*	CPIC, CPNDS	1A
Acenocoumarol	*CYP2C9*, *VKORC1*, *CYP4F2*	DPWG	1B
Simvastatin	*SLCO1B1*	CPIC	1A

**Table 2:** Main characteristics from the large scale clopidogrel studies included in this review. MACE: major adverse cardiovascular events, CV: cardiovascular, LOF: loss of function alleles, ST: stent thrombosis, PCI: percutaneous coronary intervention.

Ref	Year	Ethnic	Population Studied	*n*	PCI-Stent (%)	Follow up	Endpoint	Polymorphisms	Outcomes (LOF vs. no LOF)
**High-risk patients (PCI-stent)**									
Collet [29]	2009	Europeans	ACS	259	86	>4 years	MACE (CV death, ACS, urgent PCI)	CYP2C19*2	HR 5.38 (2.32-12.47) *p* ≤ 0.0001
							ST definite	CYP2C19*2	HR 6.04 (1.75-20.80) *p* = 0.004
Mega [22]	2009	84% Europeans	ACS stent (TRITON)	1477	100	15 months	MACE (CV death, ACS, stroke)	CYP2C19*2	HR 1.53 (1.07-2.19) *p* = 0.01
							ST definite	CYP2C19*2	HR 3.09 (1.19-8.0) *p* = 0.02
Mega [17]	2010	84% Europeans	ACS stent (TRITON)	2905	100	15 months	MACE (CV death, ACS, stroke)	CYP2C19*2 and *ABCB1*	*ABCB1* TT vs. CT/CC: HR 1.72 (1.22-2.44) *p* = 0.002 CYP2C19*2 + *ABCB1* HR 1.97 (1.38-2.82) *p* = 0.0002
Simon [30]	2009	Europeans	ACS	2208	68.7	12 months	MACE (death any cause, ACS, stroke)	*CYP2C19* and *ABCB1*	*CYP2C19*: HR 1.98 (1.10-3.58) *ABCB1*: HR 1.72 (1.20-2.47)
Sorich [31]	2010	84% Europeans	ACS stent (TRITON)	13608	100	15 months	MACE (CV death, ACS, stroke)	*CYP2C19* LOF	OR 1.63 (1.45-1.81) *p* < 0.0001
Shuldiner [32]	2009	Europeans	PCI	227	100	12 months	MACE (CV death, ACS, stroke, PCI)	CYP2C19*2	HR 2.42 (1.18-4.99) *p* = 0.02
Wallentin [33]	2010	Europeans	ACS	10285	60	12 months	MACE (CV death, ACS, stroke)	*CYP2C19*	HR at 30 days: *p* = 0.028
								*CYP2C19* and *ABCB1*	HR 1.2 (1.0-1.4) *p* = 0.047**
**Low-risk patients**									
Pare [34]	2010	Europeans-latin american	ACS stable	5059	14.5	12 months	MACE (CV death, ACS, stroke)	CYP2C19*2	*p* = 0.32

Data are showed as: OR: odds ratio, HR: hazard ratio, (95%CI), *p*-value, ** Data obtained from the frequencies of the groups.

**Table 3:** Main characteristics from the non RCT and RCT about clopidogrel included in this review. MACE: major adverse cardiovascular events, CV: cardiovascular, LOF: loss of function alleles, ST: stent thrombosis, MI: myocardial infarction.

Ref	Year	Ethnic	Population Studied	*n*	PCI-Stent (%)	Follow up	Endpoint	Polymorphisms	Outcomes (Intervention Group vs. Control Group)
**Non RCT**									
Sánchez-Ramos [35]	2016	Europeans	ACS-PCI-stent	719	100	1 year	MACE (CV death, ACS, stroke)	CYP2C19*2, *3 and *ABCB1*	HR 0.63 (0.41-0.97) *p* = 0.037 HR 0.61 (0.39-0.94) *p* = 0.02**
							ST definite	*CYP2C19* *2, *3 and *ABCB1*	HR 1.27 (0.08-20.2) *p* = 0.87
							Urgent revascularization*	CYP2C19 *2, *3 and ABCB1	HR 0.63 (0.31-1.28) *p* = 0.20
**RCT**									
Shen [36]	2016	Asians	CAD-PCI	628	100	1 month 6 months 12 months	MACE (composite of death from any cause, myocardial infarction, or target vessel revascularization)	CYP2C19*2	1.3% vs. 5.6%, *p* = 0.003 3.2% vs. 7.8%, *p* = 0.012 4.2% vs. 9.4%, *p* = 0.010
Roberts (Rapid Gene) [37]	2012	Europeans	ACS or stable angina/ stent	187	100	7 days	high on-treatment platelet reactivity	CYP2C19*2	0% vs. 30% *p* = 0.0092
Roberts (RAPID STEMI study) [38]	2016	Europeans	STEMI- stent	102	100	1 month	high on-treatment platelet reactivity	CYP2C19*2, *17 and *ABCB1* TT	OR=0.15 *p* = 0.03
Xie [39]	2013	Asians	CAD-PCI	600	100	180 days	MACE (death from any cause, MI, stroke, ischemia)	CYP2C19*2,*3	1.0% and 6.2%, *p* < 0.01
Notarangelo Pharmclo [40]	2018	Europeans	ACS	888	No data	12 months	MACE (CV death, nonfatal IM, nonfatal stroke)	CYP2C19*2, *17 and *ABCB1*	HR 0.58 (0.43-0.78) *p* < 0.001
Bergmeijer (Popular genetics) [41]	ongoing	Europeans	STEMI-stent	2500	100	15 months	MACE (CV death, ACS, stroke)	CYP2C19*2, *3	
Tailor-PCI (NCT01742117)	ongoing	Europeans	ACS or CAD/ stent	5000	100	12 months	MACE (non-fatal MI, non-fatal stroke, severe recurrent ischemia, CV death, and ST)	CYP2C19*2,*17	

Data are showed as: OR: odds ratio, HR: hazard ratio, (95%CI), p-value, * Urgent revascularization non related with ST, ** adjusted in multivariate analysis.

**Table 4:** Main characteristics from the RCT about warfarin included in this review.

Ref	Year	Ethnic	Population Studied	*n*	Follow up	Endpoint	Polymorphisms	Homozygotes Action Required	Outcomes (Intervention Group vs. Control Group)	Availability Test	Dose in Non-Genotype Group
Pirmohamed (EU-PACT) [3]	2015	2% non-European	AF (72.1%) VT (27.9%)	455	12 weeks	%TTR	CYP2C9*2,*3 *VKORC1*	*VKORC1*: 17% CYP2C9*2 and *3: 3.4%	67.4% vs. 60.3%, *p* < 0.001	2h	Fixed-dose strategy
Kimel (COAG) [79]	2015	33% non-European	AF (23%) DVT or PE (56%)	1015	4 weeks	%TTR	CYP2C9*2, *3 *VKORC1*	*VKORC1*: 11% CYP2C9*2 and *3: 1%	45.2% vs. 45% *p* = 0.91	Not before the 1^st^ dose for 55% of patients	Clinical dosing algorithm
Gage (GIFT) [82]	2017	91% European	Hip or knee arthroplasty	1650	30 and 60 days	Composite (major bleeding, INR ≥ 4, VT, death)	CYP2C9*2,*3 *CYP4F2*	NA	RR 0.73 (0.56-0.95) *p* = 0.02	NA	NA

AF: atrial fibrillation, DVT: Deep-vein thrombosis, PE: pulmonary embolism. %TTR: percentage time in therapeutic range. NA: Not applicable.
